# Amorphous Ta_*x*_Mn_*y*_O_*z*_ Layer as a Diffusion Barrier for Advanced Copper Interconnects

**DOI:** 10.1038/s41598-019-56796-y

**Published:** 2019-12-27

**Authors:** Byeong-Seon An, Yena Kwon, Jin-Su Oh, Miji Lee, Sangwoo Pae, Cheol-Woong Yang

**Affiliations:** 1 School of Advanced Materials Science and Engineering, Sungkyunkwan University, Suwon, 16419 Korea; 2 Samsung Foundry Business, Samsung Electronics, GiHeung, 17113 Korea

**Keywords:** Materials science, Nanoscience and technology

## Abstract

An amorphous Ta_*x*_Mn_*y*_O_*z*_ layer with 1.0 nm thickness was studied as an alternative Cu diffusion barrier for advanced interconnect. The thermal and electrical stabilities of the 1.0-nm-thick Ta_*x*_Mn_*y*_O_*z*_ barrier were evaluated by transmission electron microscopy (TEM) and current density–electric field (*J–E*) and capacitance–voltage (*C–V*) measurements after annealing at 400 °C for 10 h. X-ray photoelectron spectroscopy revealed the chemical characteristics of the Ta_*x*_Mn_*y*_O_*z*_ layer, and a tape peeling test showed that the Ta_*x*_Mn_*y*_O_*z*_ barrier between the Cu and SiO_2_ layers provided better adhesion compared to the sample without the barrier. TEM observation and line profiling measurements in energy-dispersive X-ray spectroscopy after thermal annealing revealed that Cu diffusion was prevented by the Ta_*x*_Mn_*y*_O_*z*_ barrier. Also, the *J–E* and *C–V* measurements of the fabricated metal-oxide-semiconductor sample showed that the Ta_*x*_Mn_*y*_O_*z*_ barrier significantly improved the electrical stability of the Cu interconnect. Our results indicate that the 1.0-nm-thick Ta_*x*_Mn_*y*_O_*z*_ barrier efficiently prevented Cu diffusion into the SiO_2_ layer and enhanced the thermal and electrical stability of the Cu interconnect. The improved performance of the Ta_*x*_Mn_*y*_O_*z*_ barrier can be attributed to the microstructural stability achieved by forming ternary Ta-Mn-O film with controlled Ta/Mn atomic ratio. The chemical composition can affect the atomic configuration and density of the Ta-Mn-O film, which are closely related to the diffusion behavior. Therefore, the 1.0-nm-thick amorphous Ta_*x*_Mn_*y*_O_*z*_ barrier is a promising Cu diffusion barrier for advanced interconnect technology.

## Introduction

A copper (Cu) interconnect can transmit clock and other signals for providing power/ground functions to various microelectronic devices. Cu interconnects require a liner/barrier to improve the adhesion between the Cu and silicon-based interlayer dielectric (ILD) materials and to block the diffusion of Cu into the ILD materials^1^. A dual Ta/TaN barrier formed by physical vapor deposition (PVD) is generally used as a diffusion barrier as it leads to good adhesion between Cu and the ILD materials, in addition to ensuring thermal stability and blocking the diffusion of Cu into the ILD material^2,3^. However, the dual Ta/TaN barrier is naturally thicker than a single barrier and can increase the electrical resistivity because it occupies a larger portion of Cu line volume in the Cu interconnect. In addition, with the scaling down expected by Moore’s Law^4^, the conventional dual Ta/TaN diffusion barrier faces technological limitations^5^, including poor step coverage by PVD methods^6^ and an inability to form a barrier layer with a thickness of 1 nm or less. Recently, thinner TaN barriers prepared by atomic layer deposition (ALD) have been investigated, but their performance as barriers is not yet perfect, making them difficult to apply in practice^7^.

Instead of a dual Ta/TaN barrier which has polycrystalline structure with inherent grain boundary diffusion pathways, an amorphous Ta_2_O_5_ barrier was investigated as a potential single barrier for Cu interconnects^8,9^. This oxide has a high thermal stability and is not reactive with Cu or SiO_2_. Salaum *et al*.^8,9^ reported that a 20-nm-thick amorphous Ta_2_O_5_ barrier showed a good performance as a diffusion barrier up to 600 °C, but it failed at higher temperatures, corresponding to the beginning of crystallization. Moreover, although amorphous Ta_2_O_5_ layers have some advantages as diffusion barriers, a Ta_2_O_5_ layer with a thickness less than 20 nm is not suitable for use as a Cu diffusion barrier because it is deposited as a discontinuous layer^8^.

A notable progress in Cu interconnects is a self-forming barrier proposed by Koike *et al*.^10^ The self-forming barrier was realized by spontaneously reacting the Cu–*X* alloying element with O and Si elements in the ILD material during post-metallization annealing^5^. Among the Cu–*X* alloying elements used^11–16^, an Mn alloying element^17,18^ is widely used because the self-formed Mn oxide or Mn silicate has good reliability and is thinner than the dual Ta/TaN barrier. The thickness of the self-forming barrier is determined by the temperature of post-metallization annealing and the type of deposition method, such as PVD, chemical vapor deposition (CVD), or ALD^19–22^. Although the CVD method yields good step coverage, Nguyen *et al*.^23^ reported that it is difficult to control the barrier thickness due to the high reactivity of the Mn precursor with the SiO_2_ layer, and thus amorphous MnSi_*x*_O_*y*_ diffusion barriers with thicknesses of less than 2.0 nm are not suitable for use as Cu diffusion barriers. Moreover, the self-forming barrier is very sensitive to the processing conditions such as temperature, annealing time, and alloying concentration.

On the other hand, a MnO_*x*_ layer formed by ALD was considered a good candidate because of its thickness controllability. However, it was found to act as a Cu diffusion barrier only when its thickness was greater than 1.2 nm^20^. Furthermore, the thickness of the ALD–MnO_*x*_ layer is affected by the adsorbed moisture on the substrate. The ALD–MnO_*x*_ layer was very thin on the hydrophobic surface of a low-k SiOCH substrate, whereas it was thicker on a TEOS–SiO_2_ substrate^24^. Thus, the surface conditions of the ILD material are very important in terms of controlling the thickness of the ALD–MnO_*x*_ layer.

An alternative Cu diffusion barrier is required to meet various requirements such as good adhesion between Cu and the SiO_2_ layer, back-end-of-line (BEOL)-compatible deposition processes, and reliability in advanced Cu interconnects^25,26^. It is desirable to avoid forming polycrystalline films with inherent grain boundaries which dominates diffusion especially at low temperatures. Therefore, the amorphous structure is still beneficial at preventing the atomic diffusion^27,28^. The incorporation of an additive element in existing transition metal oxide may offer a promising method for maintaining amorphous state by interrupting the polycrystalline phase formation. In this regard, the ternary Ta-Mn-O system is of potential interest for Cu interconnects. In addition to the ability to form an amorphous structure, the electrical resistivity of the barrier layer is another important property because it has substantial influence on the effective resistivity of the Cu interconnect. However, the resistivity of the Cu interconnect is more affected by grain boundary scattering, surface scattering, and an increasing portion of the conventional barrier in the Cu interconnect than the resistivity of the barrier layer itself^26,29^. In that sense, it is very important to minimize the thickness of the barrier layer to reduce the resistivity of the Cu interconnect. Although industry-friendly Ta-based and Mn-based barriers exhibit excellent performance in Cu interconnects, it is still challenging to fabricate a Cu diffusion barrier with a thickness less than 1.2 nm that completely prevents Cu diffusion into the ILD material.

In this study, we developed an amorphous Ta_*x*_Mn_*y*_O_*z*_ layer as an ultrathin diffusion barrier for advanced Cu interconnects that had excel`lent barrier properties, such as high thermal stability and good adhesion. A 1.0-nm-thick amorphous Ta_*x*_Mn_*y*_O_*z*_ layer was prepared by using the conventional PVD method because it allows easy and systematic investigation of the fundamental properties by changing the film thickness and chemical composition. To evaluate the thermal and electrical stability of the Ta_*x*_Mn_*y*_O_*z*_ barrier, we investigated its ability to prevent Cu diffusion into the ILD material by annealing it at 400 °C for 10 h and by applying bias thermal stress under 6 MV/cm and 150 °C for 30 min. The results of this study demonstrate that the amorphous Ta_*x*_Mn_*y*_O_*z*_ layer is a promising diffusion barrier for advanced Cu interconnects.

## Results and Discussion

**Figure 1:**
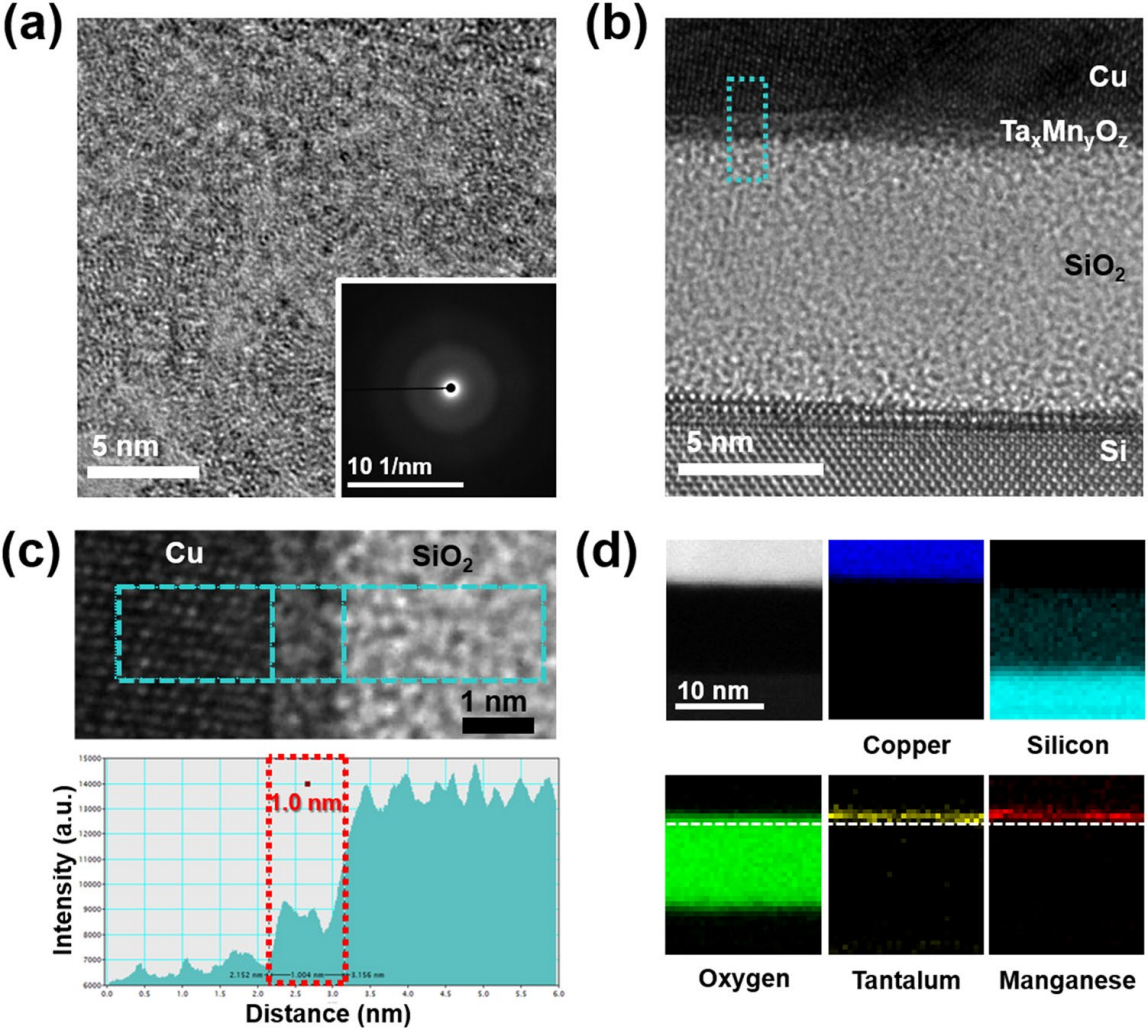
(**a**) Plan-view HR-TEM image showing the amorphous Ta_*x*_Mn_*y*_O_*z*_ layer. The SADP is shown in the inset. (b) Cross-sectional HR-TEM image of the as-deposited MOS capacitor sample with the amorphous Ta_*x*_Mn_*y*_O_*z*_ barrier. (**c**) Intensity profile for the thickness measurement of the initial Ta_*x*_Mn_*y*_O_*z*_ barrier. (**d**) STEM-EELS elemental maps of the as-deposited MOS capacitor sample with the amorphous Ta_*x*_Mn_*y*_O_*z*_ barrier.

Figure 1a shows plan-view transmission electron microscopy (TEM) images of the as-deposited Ta_*x*_Mn_*y*_O_*z*_ barrier and selected-area electron diffraction patterns (SADPs). The Ta_*x*_Mn_*y*_O_*z*_ film was in amorphous form, as seen in the plan-view TEM image, and the SADP in the inset reveals only a halo-ring pattern, indicating a perfectly amorphous structure. The thickness of the deposited Ta_*x*_Mn_*y*_O_*z*_ barrier was determined from the cross-sectional high-resolution (HR)-TEM images of the metal-oxide-semiconductor (MOS) structure with the Ta_*x*_Mn_*y*_O_*z*_ barrier and the intensity profile (Fig. 1b,c). The intensities of the Cu, the Ta_*x*_Mn_*y*_O_*z*_ barrier, and the SiO_2_ layers were different in the HR-TEM image as shown in Fig. 1c, and the Ta_*x*_Mn_*y*_O_*z*_ barrier was clearly distinguished between the Cu and SiO_2_ layer. The thickness of the barrier was 1.0 nm and it was an amorphous phase. To confirm the elemental distribution in the as-deposited MOS capacitor with the Ta_*x*_Mn_*y*_O_*z*_ barrier, the scanning TEM-electron energy loss spectroscopy (STEM-EELS) spectrum was obtained in the energy loss range for each region with Cu (L_2,3_ edge:931 eV), Ta (O_1_ edge:71 eV), Mn (L_3_ edge:640 eV), O (K edge:532 eV), and Si (K edge:99 eV). As shown in the Ta map ranging from 69.5 to 79.5 eV and the Mn map ranging from 637.5 to 647.5 eV, the Ta_*x*_Mn_*y*_O_*z*_ film was a thin layer between the Cu and SiO_2_ layers (Fig. 1d).

**Figure 2:**
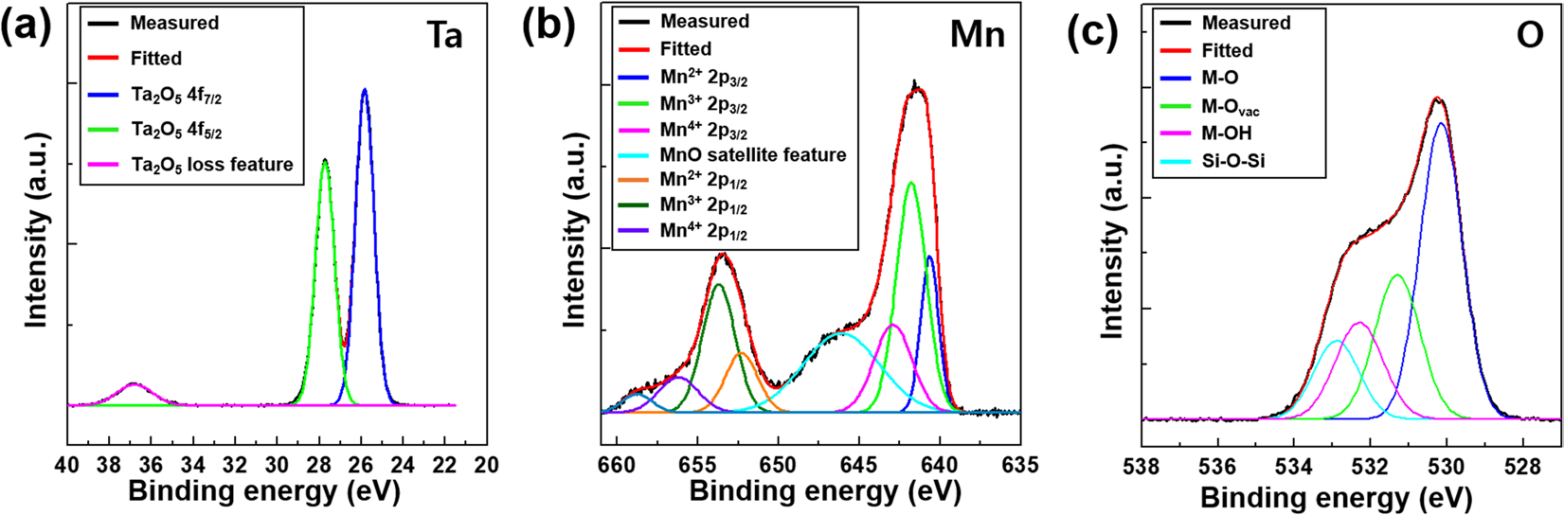
Representative XPS spectra of the as-deposited Ta_*x*_Mn_*y*_O_*z*_ barrier, (**a**) Ta 4f, (**b**) Mn 2p, and (**c**) O 1s.

To obtain the chemical information of the Ta_*x*_Mn_*y*_O_*z*_ diffusion barrier, X-ray photoelectron spectroscopy (XPS) analysis was performed and the results are shown in Fig. 2. The experimental XPS spectra were deconvoluted using Gaussian–Lorentzian peaks after background extraction. As shown in Fig. 2a, the Ta 4f spectrum revealed that Ta 4f_5/2_ and Ta 4f_7/2_ existed at peak values of 25.8 eV and 27.7 eV, respectively, and the loss feature for Ta_2_O_5_ appeared at 36.8 eV. These peaks corresponded to the binding energy of fully oxidized stoichiometric Ta_2_O_5_, which was in good agreement with previous work^30^. Fig. 2b shows the XPS spectrum of Mn 2p in the Ta_*x*_Mn_*y*_O_*z*_ diffusion barrier. The main two peaks with binding energies at 641.2 eV and 653.4 eV corresponded to Mn 2p_3/2_ and Mn 2p_1/2_, respectively. Peak fitting was conducted for Mn 2p_3/2_, which was deconvoluted into Mn^2+^, Mn^3+^, and Mn^4+^, with characteristic binding energies at 640.7 eV, 641.8 eV, and 643.1 eV, respectively^31,32^. The Mn 2p_3/2_ peak also demonstrated an MnO satellite feature at 646.2 eV. The Mn metal in the Ta_*x*_Mn_*y*_O_*z*_ barrier consisted of MnO, MnO_2_, and Mn_2_O_3_ based on the obtained binding energy at the Mn 2p_3/2_ peak. In Fig. 2c, there were three O 1 s peaks at 530.1 eV, 531.2 eV, and 532.9 eV, indicating metal–oxide (M–O), metal–oxygen vacancy (M–O_vac_), and metal–hydrogen lattice (M–OH) bonds, respectively^33^. The M–O, M–O_vac_, and M–OH bonds were formed by binding with the Ta and Mn metals in the Ta_*x*_Mn_*y*_O_*z*_ diffusion barrier.

**Figure 3:**
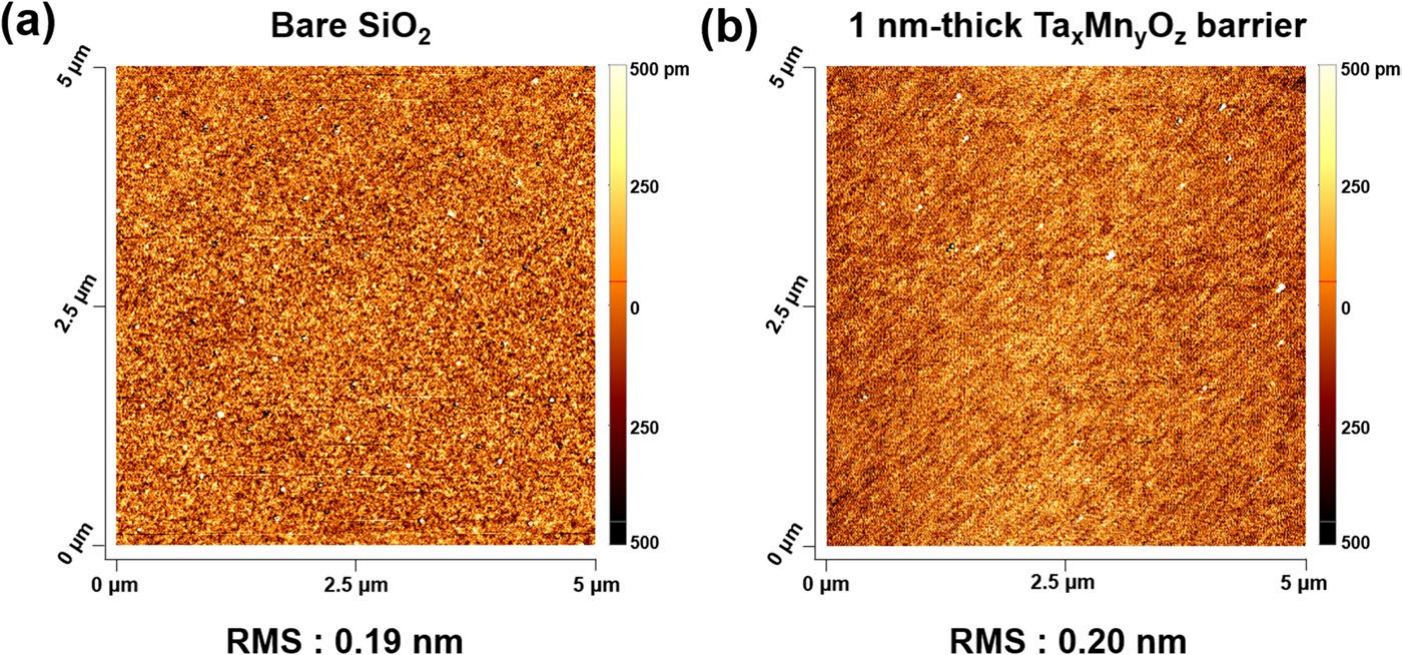
AFM images of the (**a**) bare SiO_2_ layer as a reference and the (**b**) 1.0-nm-thick Ta_*x*_Mn_*y*_O_*z*_ barrier.

**Figure 4:**
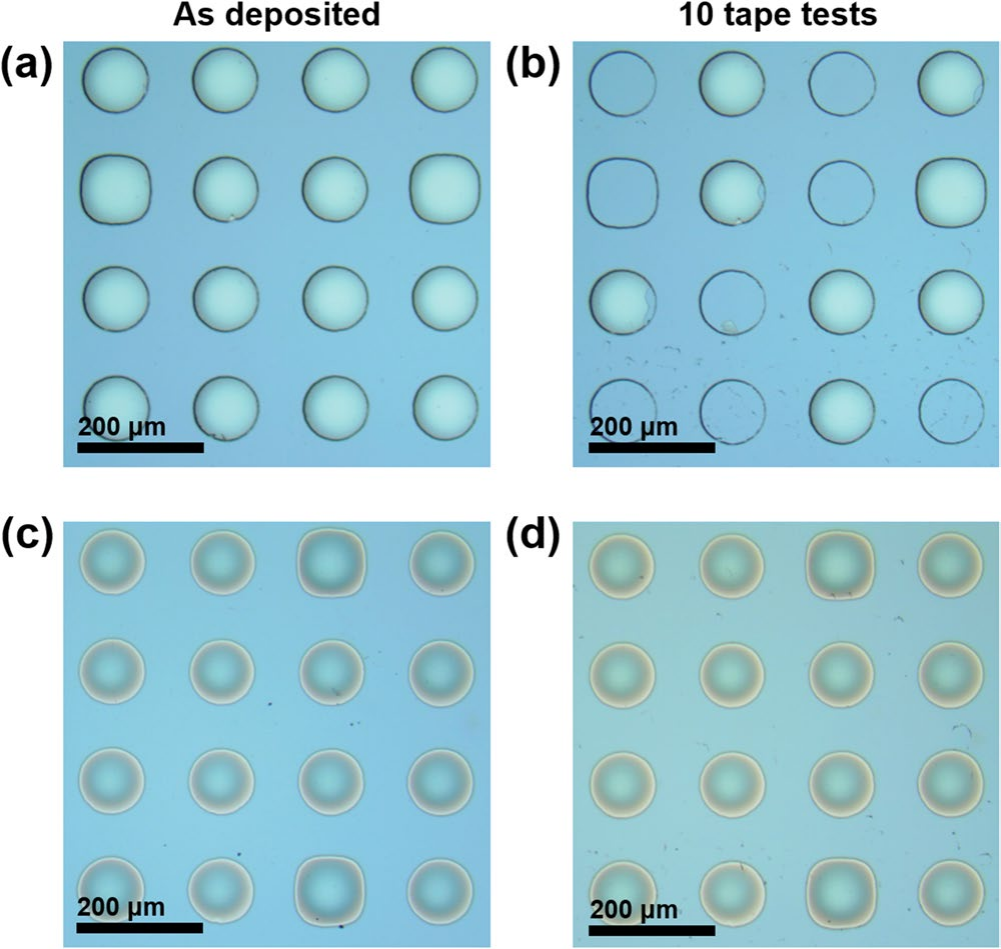
Optical microscope images of the as-deposited sample without the barrier (**a**) before the tape peeling test and (**b**) after 10 tape peeling tests, and the as-deposited sample with the amorphous Ta_*x*_Mn_*y*_O_*z*_ barrier (**c**) before the tape peeling test and (**d**) after 10 tape peeling tests.

Because the rough surface of the diffusion barrier can provide a fast diffusion pathway for Cu migration across the liner, the surface roughnesses of the 1.0-nm-thick Ta_*x*_Mn_*y*_O_*z*_ layer and the SiO_2_ layer without the barrier (reference) were measured using atomic force microscopy (AFM). A smooth surface with a root mean square (RMS) value of 0.20 nm was obtained for the 1.0-nm-thick Ta_*x*_Mn_*y*_O_*z*_ barrier, which was similar to the RMS of the 10-nm-thick SiO_2_ layer (0.19 nm), as shown in Fig. 3a,b. To examine the Cu adhesion to the SiO_2_ surface resulting from the Ta_*x*_Mn_*y*_O_*z*_ barrier, 3 M Scotch tape peeling tests were performed for the MOS capacitor samples with and without the Ta_*x*_Mn_*y*_O_*z*_ barrier. As shown in Fig. 4a,b, the MOS capacitor sample without the barrier failed the tape peeling test owing to poor adhesion, which was apparent from the removal of the Cu dot electrodes. In contrast, for the Ta_*x*_Mn_*y*_O_*z*_ barrier sample, the Cu dot electrodes deposited on the SiO_2_ layer did not change after the tape peeling test, as shown in Fig. 4c,d, despite multiple attempts to tear off the Cu dot electrodes. These results indicate that the Ta_*x*_Mn_*y*_O_*z*_ barrier had good adhesion between the Cu and SiO_2_ layers.

**Figure 5:**
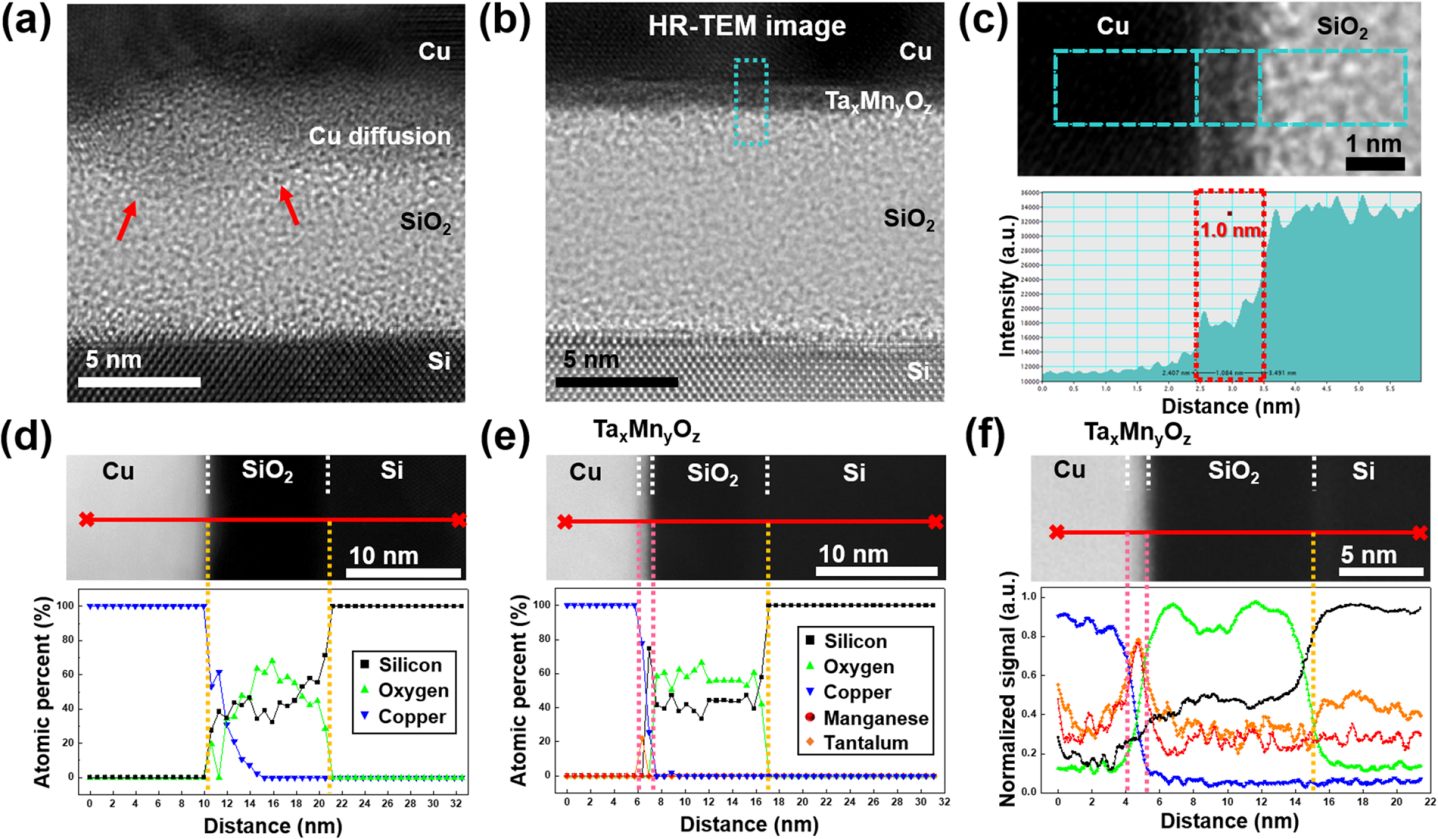
Cross-sectional HR-TEM images of the MOS capacitor sample (**a**) without the barrier and (**b**) with the Ta_*x*_Mn_*y*_O_*z*_ barrier. Image (**c**) shows the intensity profile for the thickness measurement after annealing at 400 °C for 10 h. The HAADF-STEM images and the STEM-EDS line profiles of the MOS capacitor sample are shown (**d**) without the barrier and (**e**) with the Ta_*x*_Mn_*y*_O_*z*_ barrier after annealing at 400 °C for 10 h. Image (**f**) shows the HAADF-STEM image and the STEM-EELS line profile of the MOS capacitor sample with Ta_*x*_Mn_*y*_O_*z*_ barrier after annealing at 400 °C for 10 h.

To investigate the thermal stability of the 1.0-nm-thick Ta_*x*_Mn_*y*_O_*z*_ barrier, we annealed the MOS capacitor samples with and without the Ta_*x*_Mn_*y*_O_*z*_ barrier at 400 °C for 10 h. Figure 5 shows the cross-sectional HR-TEM images and the line profile of the chemical composition for each element after annealing at 400 °C for 10 h. To compare the results with and without the Ta_*x*_Mn_*y*_O_*z*_ barrier, a MOS capacitor sample without a barrier was prepared as a reference. For the sample without the Ta_*x*_Mn_*y*_O_*z*_ barrier, the interface between the Cu and SiO_2_ layer showed a noticeable difference because of Cu diffusion into the SiO_2_ layer during annealing, as shown in Fig. 5a. The initial SiO_2_ layer with a thickness of 10 nm was reduced to approximately 5.8 nm owing to Cu diffusion. Conversely, the annealed sample with the Ta_*x*_Mn_*y*_O_*z*_ barrier was identical to the initial interface between the Cu and SiO_2_ layers and had a uniform thickness of 1.0 nm, which was consistent with the as-deposited Ta_*x*_Mn_*y*_O_*z*_ barrier before annealing, as shown in Fig. 5b,c. In addition, the STEM-energy dispersive X-ray spectroscopy (STEM-EDS) line profile, shown in Fig. 5d,e, was used to accurately evaluate the Cu diffusion. For the sample without the Ta_*x*_Mn_*y*_O_*z*_ barrier (see Fig. 5d), Si and O were detected in the SiO_2_ layers, as shown in the high-angle annular dark-field (HAADF) STEM image, and the distribution of Cu showed that the Cu atoms diffused into the SiO_2_ layer. The annealed Ta_*x*_Mn_*y*_O_*z*_ diffusion barrier showed the same chemical binding states as the as-deposited Ta_*x*_Mn_*y*_O_*z*_ barrier, as shown in Fig. S1 of the SI. In the STEM-EDS line profile of the annealed sample with the Ta_*x*_Mn_*y*_O_*z*_ barrier shown in Fig. 5e, Cu was not detected inside the SiO_2_ layer, which indicated no diffusion of Cu into the SiO_2_ layer. However, the Ta and Mn in the diffusion barrier were not properly detected in the barrier region between the Cu and the SiO_2_ layers, despite the presence of the Ta_*x*_Mn_*y*_O_*z*_ barrier. In fact, the quantitative analysis using STEM-EDS for Ta in the MOS capacitor sample was difficult because the Ta Mα (1.71 keV) and Ta Lα (8.14 keV) X-ray energies overlapped with the Si Kα (1.74 keV) and Cu Kα (8.04 keV) X-ray energies, respectively. In addition, Mn was difficult to detect in the line profile owing to the damage caused by the 200-keV e-beam during the STEM-EDS analysis. To complement STEM-EDS, Ta and Mn were confirmed using STEM-EELS. As shown in Fig. 5f, Ta and Mn were detected at the interface between the Cu and SiO_2_ layers. In addition, the Ta_*x*_Mn_*y*_O_*z*_ barrier prevented the diffusion of Cu toward the SiO_2_ layer, which was consistent with the Cu distribution in the STEM-EDS line profile. However, the detection of trace elements that may have affected the device characteristics was difficult because the detection limits of STEM-EDS and -EELS are approximately 0.1–3.0 wt.%^34^. Therefore, to determine the presence of a few Cu atoms in amounts as low as 10^9^ atoms/cm^3^ ^35^, the current density-electric field (*J–E*) and capacitance-voltage (*C–V*) characteristics were evaluated.

**Figure 6:**
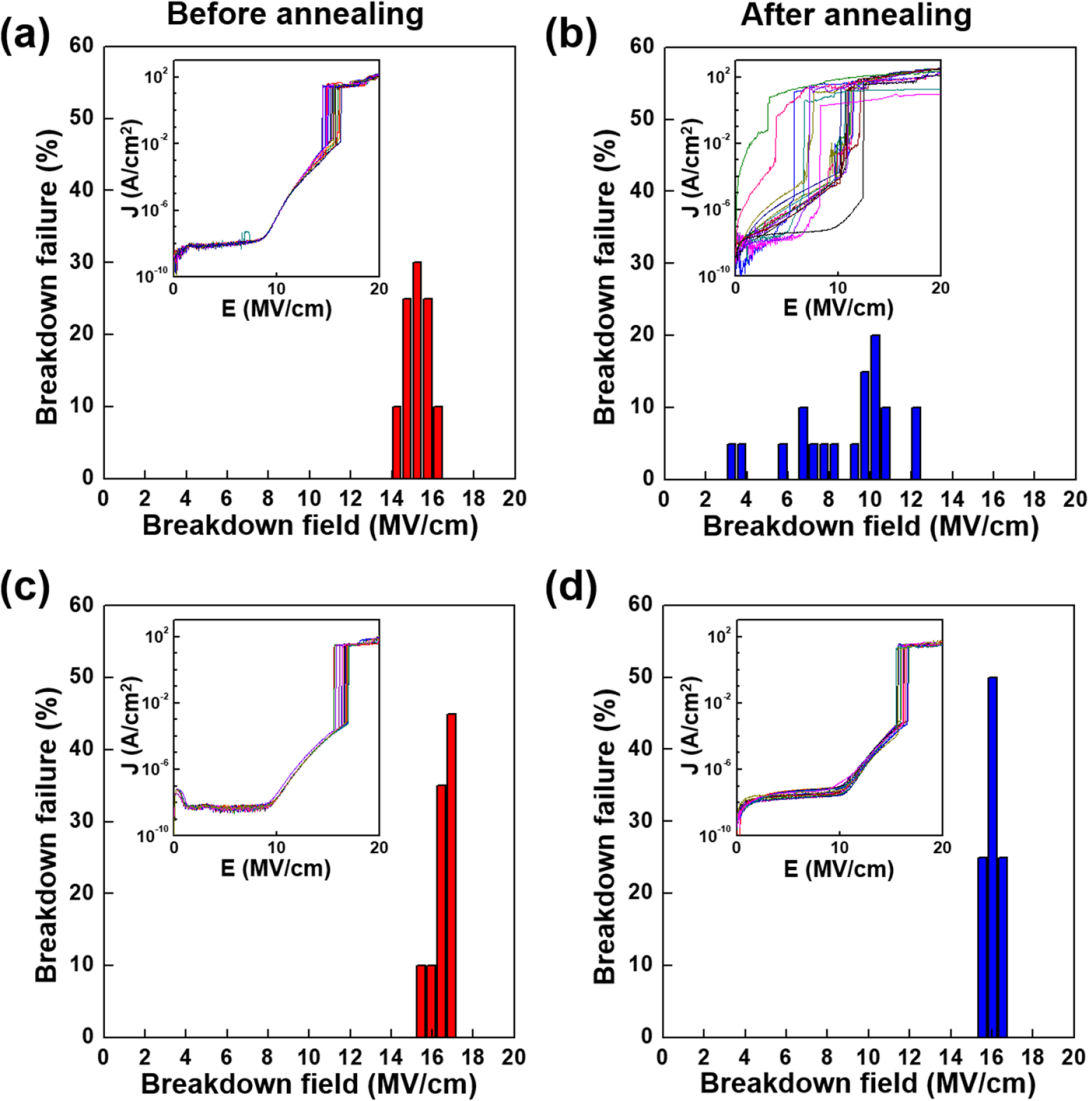
TZDB histogram obtained using 20 *J–E* curves for the evaluation of Cu diffusion into the SiO_2_ layer following annealing at 400 °C for 10 h. The MOS capacitor sample without the barrier (*a*) before and (*b*) after annealing and with the Ta_*x*_Mn_*y*_O_*z*_ barrier (*c*) before and (*d*) after annealing.

Figure 6 shows the dielectric breakdown field of a statistical time-zero dielectric breakdown (TZDB) histogram obtained using 20 *J–E* curves, as shown in the inset of Fig. 6 for the as-deposited and annealed MOS capacitor samples. In the statistical results of the TZDB histogram, three modes, referred to as A-, B-, and C-modes, were observed when the breakdown fields were composed of low (<1 MV/cm), intermediate, and high (>14 MV/cm) fields, respectively^36^. The A- and B-mode failures could be attributed to localized defect spots, such as Cu^+^ ion transport, referred to as an extrinsic breakdown, and the C-mode failure corresponded to an approximately defect-free oxide sample, was referred to as an intrinsic breakdown. As shown in Fig. 6a,b, the MOS capacitor sample without the barrier exhibited the C-mode failure ranged from 14.25 MV/cm to 16.25 MV/cm before annealing, whereas the A- and B-mode failures ranged from 3.25 MV/cm to 12.25 MV/cm after annealing at 400 °C for 10 h. Thus, the MOS capacitor sample without the barrier exhibited extrinsic breakdown because of the Cu^+^ ions that diffused into the SiO_2_ layer during annealing. Conversely, in Fig. 6c,d, the Ta_*x*_Mn_*y*_O_*z*_ barrier samples exhibited C-mode failure regardless of annealing, unlike the reference sample without the barrier, and the breakdown field before and after thermal annealing ranged from 15.45 MV/cm to 16.95 MV/cm and from 15.55 MV/cm to 16.55 MV/cm, respectively. These results provide evidence that the penetration of Cu^+^ ions into the SiO_2_ layer did not occur, which is consistent with the TEM results shown in Fig. 5.

In addition, a correlative analysis of the XPS and *J–E* measurements was carried out to evaluate the performance of the 1.0-nm-thick Ta_*x*_Mn_*y*_O_*z*_ barrier layer according to the content of Ta and Mn. The Ta/Mn ratio (i.e., the normalized chemical composition of the metals) of the as-deposited Ta_*x*_Mn_*y*_O_*z*_ layer was estimated by the wide-scan XPS survey spectra, and the TZDB histogram was measured to evaluate the Cu-blocking capability of the Ta_*x*_Mn_*y*_O_*z*_ barrier after annealing at 400 °C for 10 h. Three 1.0-nm-thick Ta_*x*_Mn_*y*_O_*z*_ barriers, each with a different Ta/Mn ratio, were prepared by controlling only the deposition time during DC sputtering. As shown in Fig. S2 of the SI, the 1.0-nm-thick Ta_*x*_Mn_*y*_O_*z*_ barrier with a normalized Ta content of 91.5% (balance Mn) could not completely block Cu diffusion into the SiO_2_ layer during annealing. Conversely, for the Ta_*x*_Mn_*y*_O_*z*_ barrier with normalized Ta content range from 56.5% to 62.6%, the TZDB results showed that the penetration of Cu^+^ ions into the SiO_2_ layer did not occur. Therefore, the chemical composition (Ta/Mn ratio) can affect the performance of Ta_*x*_Mn_*y*_O_*z*_ barrier. The incorporation of an additive element in existing binary system can lead to a change in atomic configuration and density of the barrier and contribute to maintain a high thermal stability by suppressing the polycrystalline phase formation^37–39^. Fig. S3 of the SI shows the detailed Ta 4f XPS spectrum of Ta_*x*_Mn_*y*_O_*z*_ barrier with the normalized Ta content of 91.5% in Fig. S2a. This deconvoluted Ta 4f XPS spectrum revealed that the Ta 4f_7/2_ and 4f_5/2_ binding energies corresponded to the metallic Ta as well as the amorphous Ta_2_O_5_, unlike Ta_*x*_Mn_*y*_O_*z*_ barrier containing the normalized Ta content of 46.55% (Fig. 2a). Therefore, the poor barrier property of the Ta_*x*_Mn_*y*_O_*z*_ layer with a high Ta/Mn ratio can be attributed to a lack of Mn and O atoms to bind Ta and the presence of metallic Ta atoms. Bassiri *et al*.^40^ reported that the Ta-Ta and Ta-O bonding distances were determined as 3.1 Å and less than 2 Å, respectively, by analyzing high-quality EXAFS spectra of ion beam sputtered amorphous tantala. This implies that the amount of Ta-Ta and Ta-O bonds is closely related to the atomic configuration and density of the Ta_*x*_Mn_*y*_O_*z*_ film. Therefore, the performance of 1.0 nm-thick Ta_*x*_Mn_*y*_O_*z*_ barrier can be affected by the binding states between Ta, Mn and O, depending on the Ta/Mn ratio.

**Figure 7:**
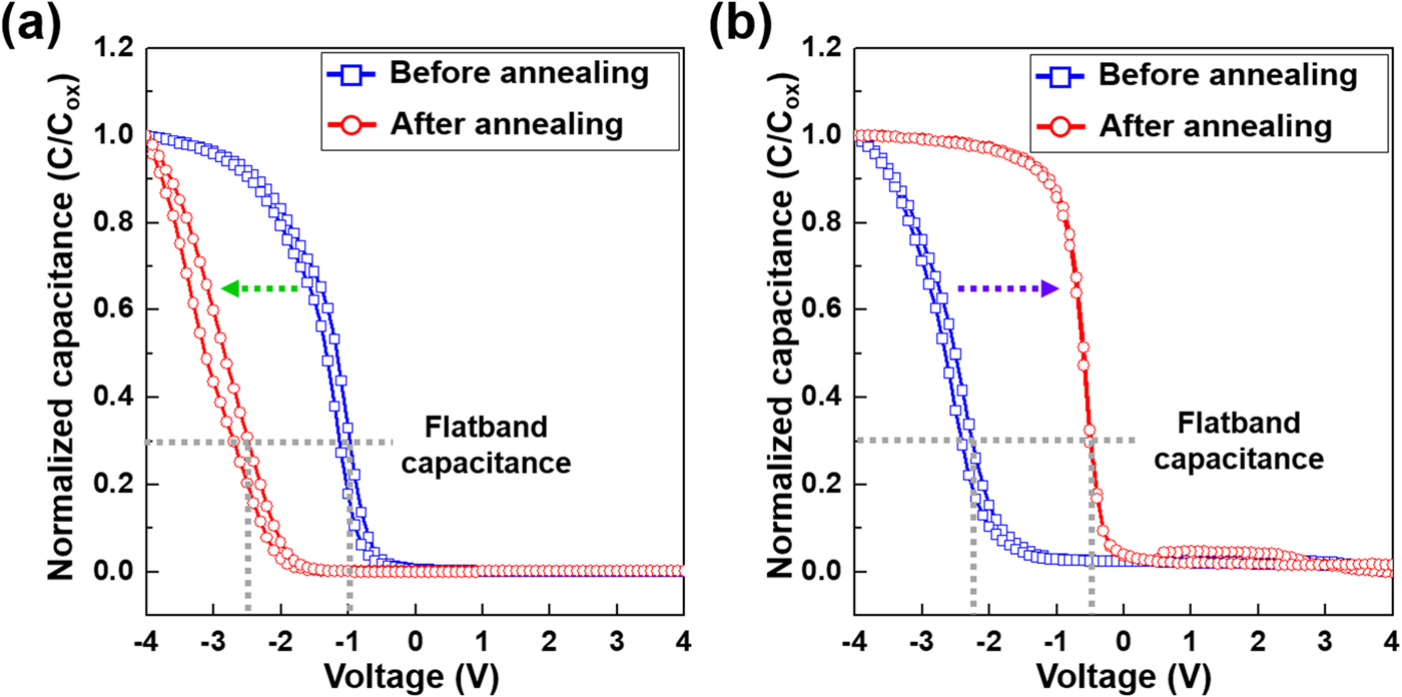
Normalized *C–V* measurements of the MOS capacitor sample (**a**) without the barrier and (**b**) with the 1.0 nm thick Ta_*x*_Mn_*y*_O_*z*_ barrier for the thermal stability evaluation.

The normalized *C–V* characteristics of MOS capacitor samples with and without the Ta_*x*_Mn_*y*_O_*z*_ barrier upon annealing at 400 °C for 10 h are shown in Fig. 7. In Fig. 7a, the MOS capacitor sample without the barrier shows a negative flatband voltage shift (indicated by the green arrow) from −0.94 V to −2.54 V upon annealing owing to Cu^+^ ion transport into the SiO_2_ layer, which indicates a buildup of positive charge within the SiO_2_ layer during annealing^18^. In addition, the hysteresis in the *C–V* curve indicates movement of the Cu^+^ ion within the dielectric film during the *C–V* sweep. It was noted that the flatband voltage of the MOS capacitor with the Ta_*x*_Mn_*y*_O_*z*_ barrier before annealing was more negative than that of the as-deposited reference sample without the Ta_*x*_Mn_*y*_O_*z*_ barrier (Cu/SiO_2_/Si). This can be attributed to intrinsic defects (positive charge was calculated as 4.63 × 10^−11^ C) such as charges in SiO_2_ or oxygen vacancies in the Ta_*x*_Mn_*y*_O_*z*_ layer^18,41–43^. The flatband voltage moved to the positive region from −2.25 V to −0.49 V (indicated by the purple arrow) upon annealing owing to the removal of the intrinsic defects in the MOS capacitor sample and not from the diffusion of Cu^+^ ions into the SiO_2_ layer^18^. In other words, there was no flatband voltage shift toward the negative region, and no hysteresis curve was observed in the MOS capacitor with the Ta_*x*_Mn_*y*_O_*z*_ barrier, as shown in Fig. 7b, indicating that the Cu^+^ ions were effectively prevented from diffusing into the SiO_2_ layer by the Ta_*x*_Mn_*y*_O_*z*_ barrier.

**Figure 8:**
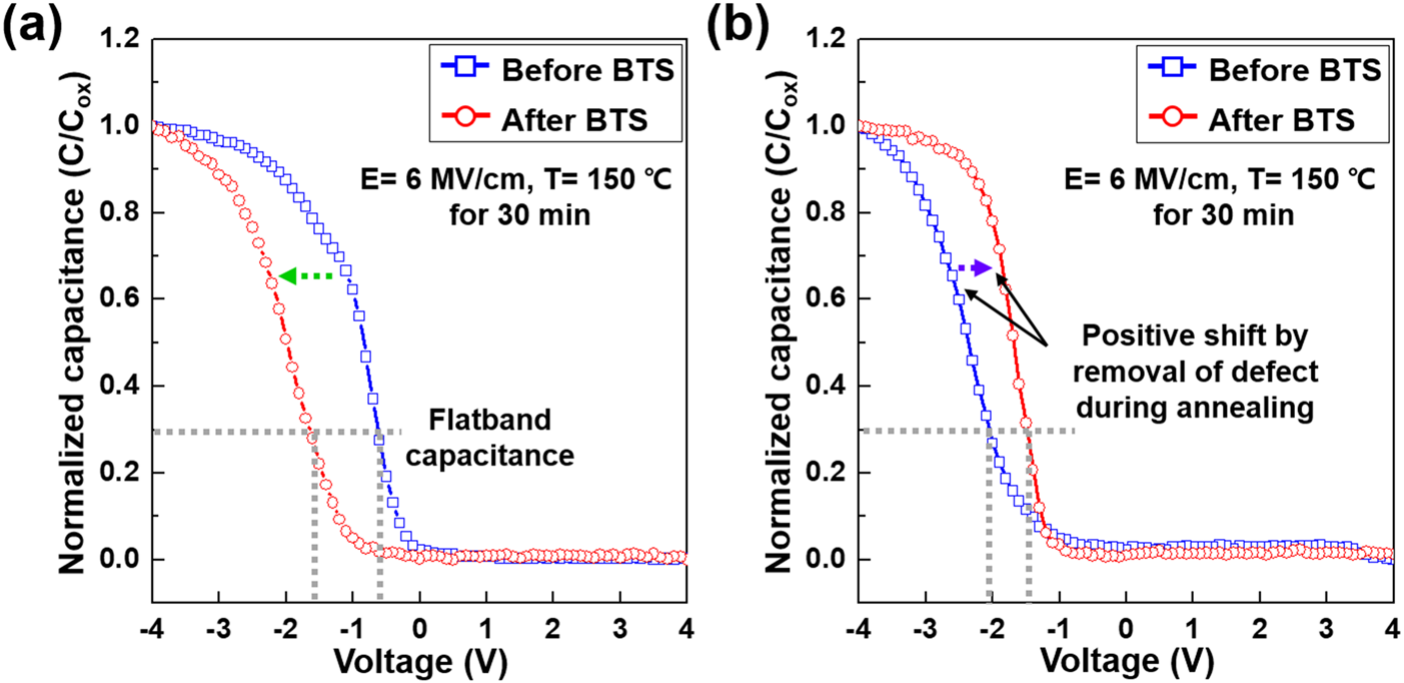
Normalized *C–V* measurements of the MOS capacitor sample (**a**) without the barrier and (**b**) with the 1.0 nm thick Ta_*x*_Mn_*y*_O_*z*_ barrier for the electrical stability evaluation under bias thermal stress.

The normalized *C–V* characteristics before and after bias thermal stress (BTS) were also measured. Figure 8 shows the normalized *C–V* curves for MOS capacitor with and without the Ta_*x*_Mn_*y*_O_*z*_ barrier after BTS at 6 MV/cm and 150 °C for 30 min. As shown in Fig. 8a, for the MOS capacitor without a barrier, the normalized *C–V* curve presented a roughly −1.0 V observable shift of flatband voltage toward the negative region under an electrical field of 6 MV/cm, which is consistent with the normalized *C–V* sweep after thermal annealing. Conversely, the MOS capacitor with the Ta_*x*_Mn_*y*_O_*z*_ barrier showed no negative shift in flatband voltage, as shown in Fig. 8b. A positive shift of flatband voltage in the *C–V* curve occurred because of the removal of intrinsic defects during annealing.

Consequently, these results provide direct evidence that the 1.0-nm-thick amorphous Ta_*x*_Mn_*y*_O_*z*_ layer is suitable for use as an ultrathin diffusion barrier that can improve the reliability and lifetime of advanced Cu interconnects.

## Conclusions

We investigated the effectiveness of an amorphous Ta_*x*_Mn_*y*_O_*z*_ layer as an ultrathin diffusion barrier for advanced Cu interconnects. A 1.0-nm-thick Ta_*x*_Mn_*y*_ layer was oxidized by exposure to air to obtain an amorphous Ta_*x*_Mn_*y*_O_*z*_ film. Ta and Mn were present as Ta_2_O_5_, MnO, MnO_2_, and Mn_2_O_3_ in the amorphous Ta_*x*_Mn_*y*_O_*z*_ film. The tape peeling test for the MOS capacitor sample showed no delamination in the presence of the Ta_*x*_Mn_*y*_O_*z*_ layer, which improved the adhesion between the Cu and the SiO_2_ layers. To evaluate the diffusion barrier properties of the 1.0-nm-thick amorphous Ta_*x*_Mn_*y*_O_*z*_ film, MOS capacitor samples with and without the barrier were annealed at 400 °C for 10 h. HR-TEM and STEM-EDS/EELS analyses showed that the amorphous Ta_*x*_Mn_*y*_O_*z*_ barrier had a stable microstructure and chemical composition, even after thermal annealing. The *J–E*, *C–V* and TZDB results showed that the diffusion of Cu was effectively blocked by the amorphous Ta_*x*_Mn_*y*_O_*z*_ barrier. The correlative analysis of XPS and TZDB revealed that, with a comparable O concentration, the Ta/Mn atomic ratio affected the atomic configuration and density of Ta–Mn–O films. To prevent the Cu diffusion into the Ta–Mn–O film, the Ta/Mn ratio needs to be high to ensure a high atomic density. However, the Ta/Mn ratio cannot be too high because a lack of Mn and O atoms to bind Ta and the presence of the metallic Ta atoms deteriorates the barrier performance. In conclusion, a 1.0-nm-thick amorphous Ta_*x*_Mn_*y*_O_*z*_ layer serves as an adhesion promotor and excellent diffusion barrier, and it can be used as a single liner/barrier material for advanced Cu interconnects.

## Methods

The substrates for barrier deposition were 10-nm-thick SiO_2_ films on p-type doped silicon (100) wafers and were cleaned using a piranha solution of 3:1 sulfuric acid (H_2_SO_4_) and hydrogen peroxide (H_2_O_2_). Then, the Mn layer was deposited first, followed by Ta layer deposition on the SiO_2_/Si substrate by direct current (DC) magnetron sputtering using a Mn target (purity 99.9%) and Ta target (purity 99.99%) under a power of 5W at an Ar gas pressure of 5.5 mTorr (see Fig. S4 of the SI for the deposition process and the formation of the Ta_*x*_Mn_*y*_O_*z*_ layer). The DC sputtering chamber was pumped down to a base pressure less than 4.0 × 10^−7^ Torr and pre-sputtering was conducted at 10 W higher than the processing power for 20 min before deposition. The sequentially deposited ultrathin Mn and Ta films were intermixed to form a Ta_*x*_Mn_*y*_ film. The 1.0-nm-thick Ta_*x*_Mn_*y*_ film was oxidized by atmospheric exposure for the *ex-situ* sputtering to deposit the Cu film. Fig. S4 of the SI shows a schematic of the oxidation mechanism of the Ta_*x*_Mn_*y*_ film. The oxidized Ta_*x*_Mn_*y*_ film is referred to as the Ta_*x*_Mn_*y*_O_*z*_ layer. It was found that this intermixing and oxidation mechanism was valid only for sequentially deposited ultrathin Mn and Ta films with a total thickness less than 2.5 nm (see Fig S5 of the SI).

To fabricate the MOS capacitor structure, 150-nm-thick Cu films were then deposited on the Ta_*x*_Mn_*y*_O_*z*_ film by DC magnetron sputtering with dots of 100 µm diameter through a shadow mask, and a 50-nm-thick Ta film was coated as the capping layer. MOS capacitor samples with and without the 1.0-nm-thick Ta_*x*_Mn_*y*_O_*z*_ layer were prepared using the same deposition method. To evaluate the thermal stability of the Ta_*x*_Mn_*y*_O_*z*_ layer as a diffusion barrier, the as-deposited MOS capacitor samples were annealed at 400 °C for 10 h in a tube furnace under Ar + 10% H_2_ gas flow. XPS (ESCALAB250Xi, Thermo-Scientific, UK) was used to determine the chemical characteristics of the deposited Ta_*x*_Mn_*y*_O_*z*_ layer. Samples for cross-sectional transmission electron microscopy (TEM) were fabricated using a focused ion beam (FIB, NX2000, Hitachi). The TEM samples were milled using a high-energy Ga^+^ ion beam from 30 keV to 5 keV and a low-energy Ar^+^ ion beam of 1 keV after the electron beam-induced deposition of the W material as a protective layer to minimize damage to the surface layers during the FIB milling process^44,45^. TEM (JEOL ARM-200F) analysis at 200 kV with EDS and EELS were used to analyze the microstructure and to obtain the line profiles for the chemical composition of the Cu, Ta, Mn, Si, and O elements in the two types of samples before and after annealing. The surface morphology of the 1.0-nm-thick Ta_*x*_Mn_*y*_O_*z*_ layer was analyzed by AFM (Park System Co.), and the adhesion strength between the Cu and SiO_2_ layers depending on the presence of the Ta_*x*_Mn_*y*_O_*z*_ barrier was examined using a 3M Scotch tape peeling test^46^.

To evaluate the Cu diffusion barrier performance of Ta_*x*_Mn_*y*_O_*z*_ after annealing at 400 °C for 10 h, the *J–E* characteristics were evaluated using a microprobe system connected to an Agilent B1500A parametric analyzer. The *J–E* curves were measured by applying voltage ranging from 0 V to −20 V in −50-mV steps. The BTS tests were performed by using a microprobe system capable of annealing and applying a high electric field to the MOS capacitor in a controlled environment. For the BTS tests, a bias field of 6 MV/cm was applied on the MOS capacitor at 150 °C for 30 min. After a given time period, the samples were cooled to room temperature. The *C–V* characteristics before and after thermal annealing and BTS were evaluated using a probe station with an Agilent E4980A precision LCR meter with an AC frequency of 100 kHz. For all *C–V* measurements, a voltage ranging from +4 V to −4 V was applied in 0.1-V steps, and the hysteresis was scanned by applying a reverse voltage of −4 V to +4 V.

## Supplementary information

Supplementary Information.
