# Supplementary material for: Amorphous TaxMnyOz Layer as a Diffusion Barrier for Advanced Copper Interconnects
Source: Sci Rep. 2019 Dec 27;9:20132. doi: 10.1038/s41598-019-56796-y (PMC6934869; doi:10.1038/s41598-019-56796-y)
Supplement: Supplementary file 1 — Supplementary Information. [file 41598_2019_56796_MOESM1_ESM.pdf]

Supplementary Information

Amorphous $Ta_xMn_yO_z$ Layer as a Diffusion Barrier for Advanced Copper Interconnects

Byeong-Seon An[1], Yena Kwon[1], Jin-Su Oh[1], Miji Lee[2], Sangwoo Pae[2] and Cheol-Woong Yang[1]*

1 School of Advanced Material Science and Engineering, Sungkyunkwan University, Suwon 16419, Korea

2 Samsung Foundry Business, Samsung Electronics, Gi-Heung 17113, Korea

*Correspondence to: cwyang@skku.edu

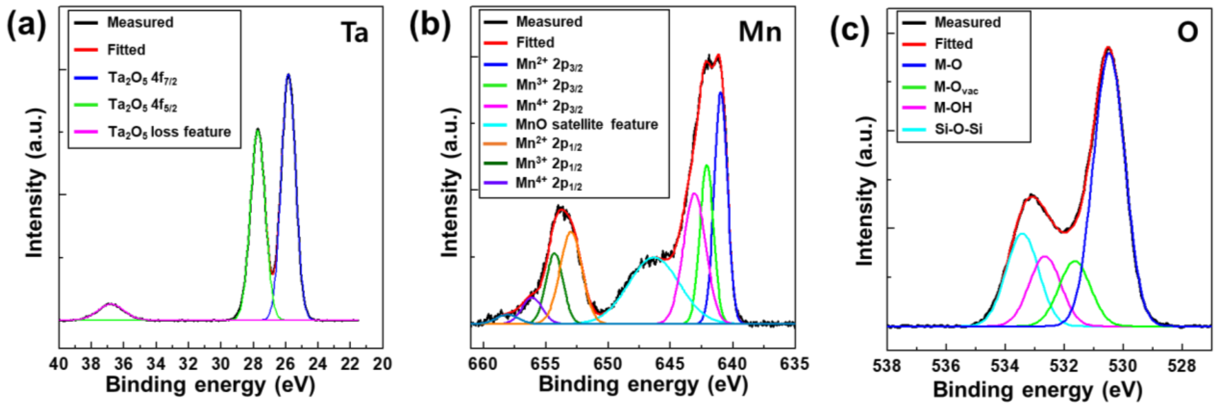

Figure S1. Representative XPS spectra of the 400 °C annealed $Ta_xMn_yO_z$ barrier: (a) Ta 4f, (b) Mn 2p, and (c) O 1s. The annealed $Ta_xMn_yO_z$ barrier shows the same chemical binding states as the as-deposited $Ta_xMn_yO_z$ barrier. However, the area fraction of the M–O_{vac} peak was markedly reduced after annealing. This indicates that oxygen vacancies in the $Ta_xMn_yO_z$ barrier were removed or mitigated during annealing.

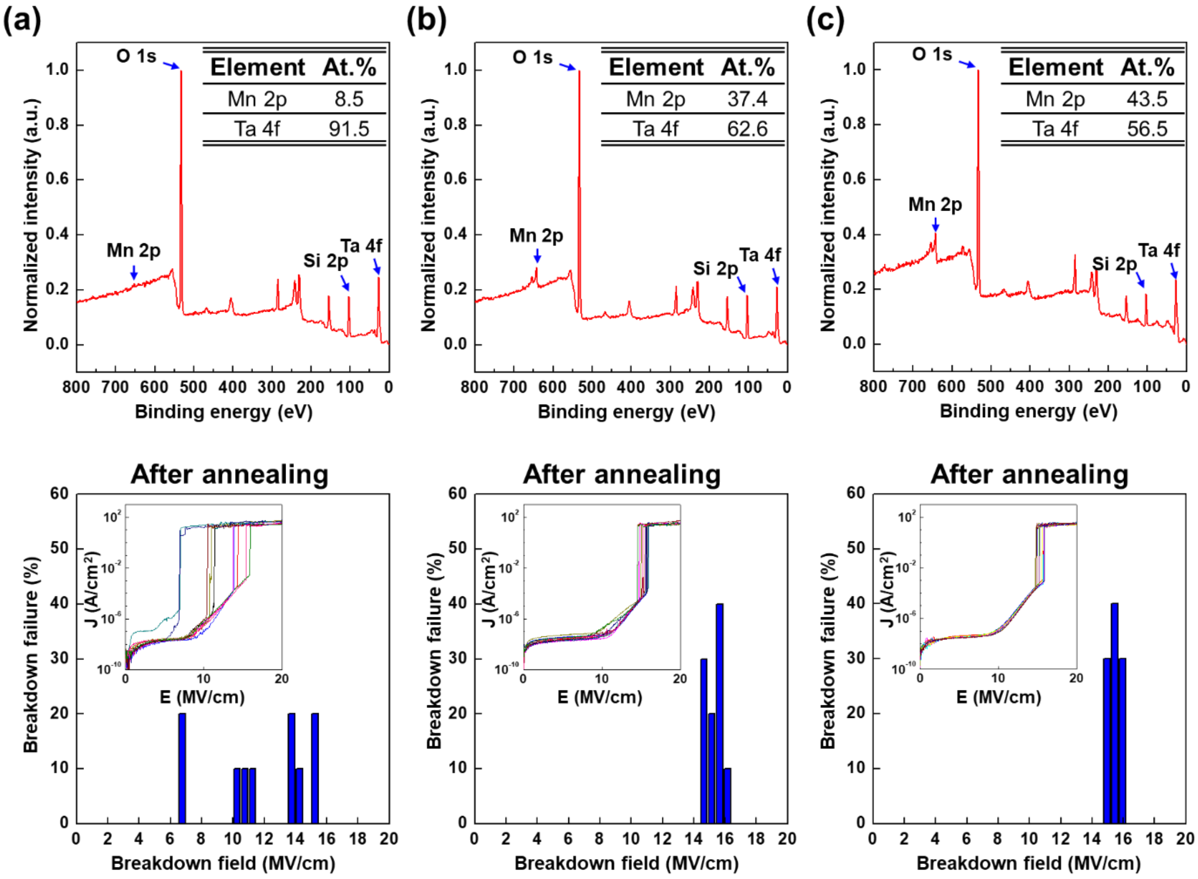

Figure S2. Wide-scan XPS survey spectra to determine the chemical composition of the $Ta_xMn_yO_z$ barrier and the results of TZDB measurements obtained using *J–E* curves for the evaluation of Cu-blocking capability during thermal annealing at 400 °C for 10 h according to the change in the normalized content of Ta and Mn (Ta/Mn ratio) in the 1.0-nm-thick $Ta_xMn_yO_z$ barrier. MOS structures with $Ta_xMn_yO_z$ barriers containing normalized Ta contents of (a) 91.5%, (b) 62.6%, and (c) 56.5% were prepared by controlling the deposition time of each element during DC sputtering. In the statistical results of the TZDB measurement, the $Ta_xMn_yO_z$ barrier with a Ta content of 91.5% showed that the A- and B-mode failures ranged from 6.75 to 15.25 MV/cm after annealing at 400 °C for 10 h. This implies that the $Ta_xMn_yO_z$ barrier with a high Ta content of 91.5% could not completely block Cu diffusion into the SiO_2 layer. Conversely, the $Ta_xMn_yO_z$ barrier with a Ta content ranging from 62.6 to 56.5% showed C-mode failure regardless of annealing. This provides

evidence that the penetration of Cu^+ ions into the SiO_2 layer did not occur. Consequently, changes in the chemical composition can affect the performance of a $Ta_xMn_yO_z$ barrier.

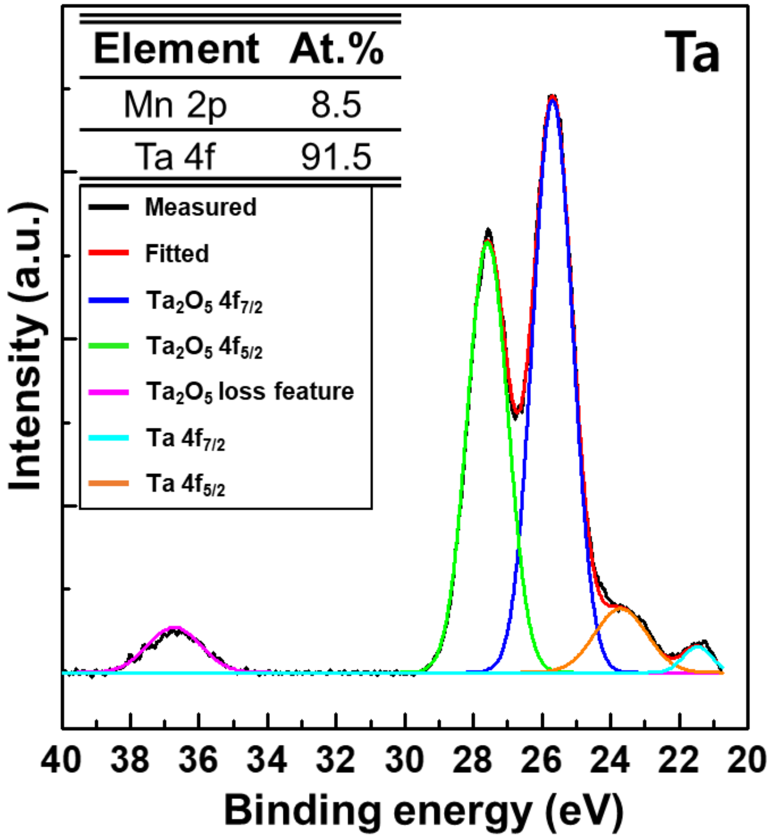

Figure S3. Ta 4f XPS spectrum of as-deposited $Ta_xMn_yO_z$ barrier with the normalized Ta content of 91.5 % in Figure S2a. This deconvoluted Ta 4f spectrum revealed that the Ta $4f_{7/2}$ and $4f_{5/2}$ binding energy existed at the peak values of 21.48 and 23.68 eV related to metallic Ta, as well as Ta $4f_{7/2}$ (25.68 eV) and $4f_{5/2}$ (27.58 eV) peak related to Ta_2O_5. The XPS result indicates that the presence of metallic Ta in 1.0 nm-thick $Ta_xMn_yO_z$ barrier is responsible for the poor barrier properties as shown in Figure S2a.

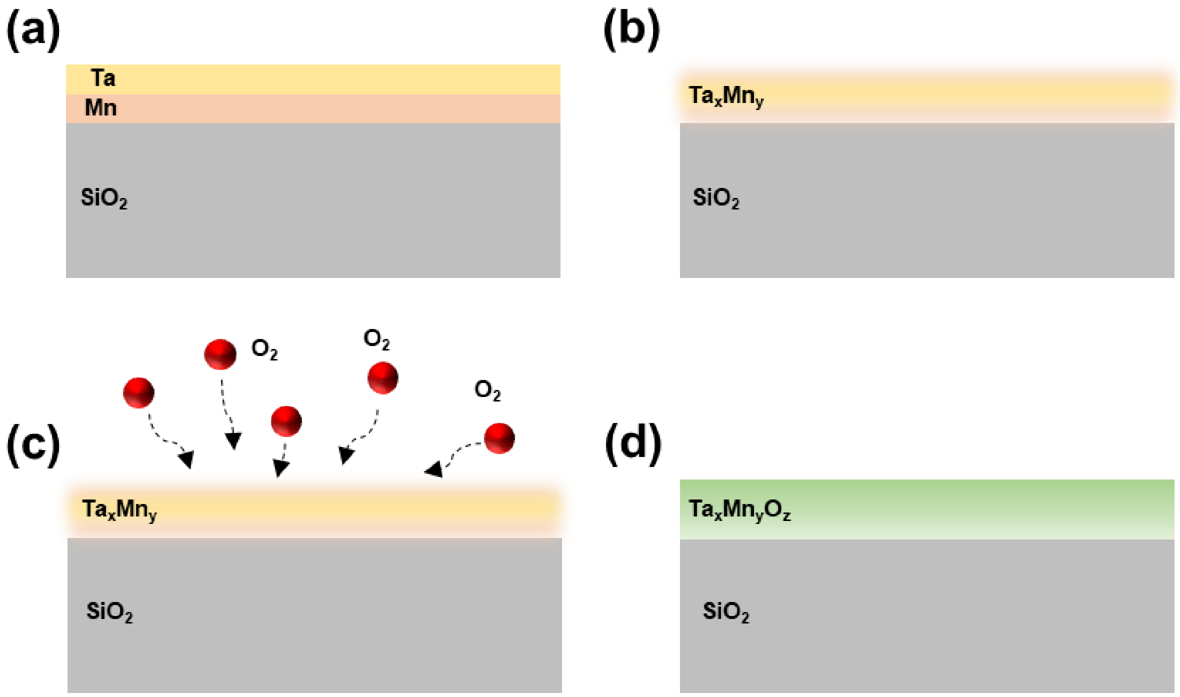

Figure S4. Schematic of the deposition process and oxidation mechanism of the Ta_xMn_y film. An oxidation mechanism of the $Ta_xMn_yO_z$ barrier can be proposed based on experimental observation. Ideally, the ultrathin Ta/Mn double layer should be separated because each Mn and Ta layer is deposited sequentially (a). However, the ultrathin Ta/Mn double layer is actually intermixed to form a single Ta_xMn_y layer (b). The Ta_xMn_y layer with thickness up to 2.5 nm is oxidized by atmospheric exposure for the ex-situ sputtering to deposit the Cu films (c). The oxidized Ta_xMn_y film is referred to as the $Ta_xMn_yO_z$ layer (d).

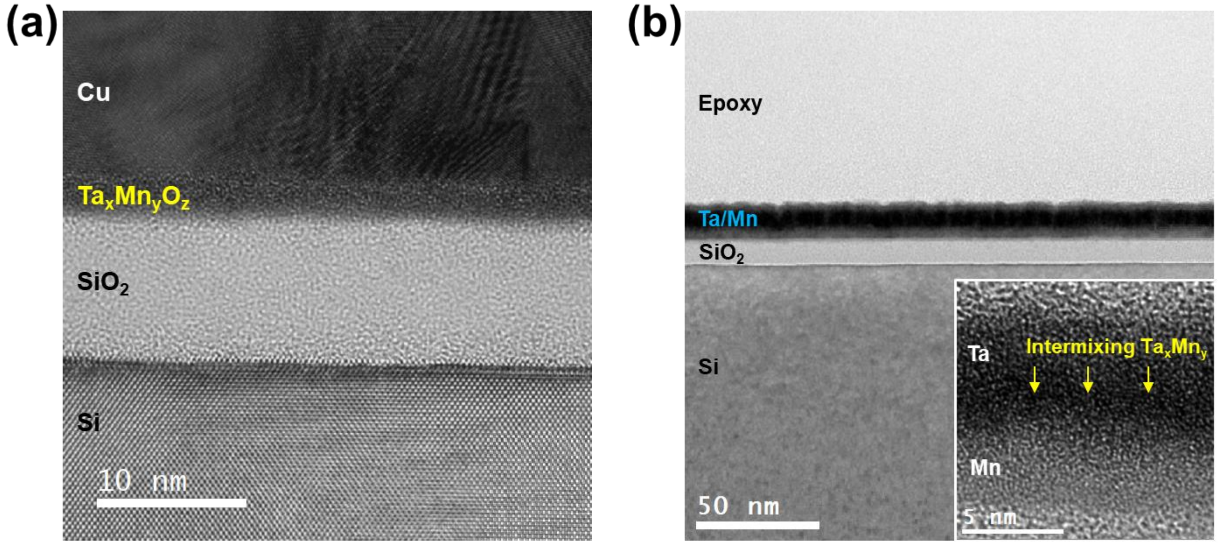

Figure S5. Cross-sectional HR-TEM images of (a) 2.5-nm-thick $Ta_xMn_yO_z$ layer and (b) 10-nm-thick Ta/Mn double layer showing the microstructural evolution of the sequentially deposited Ta/Mn double layer according to thickness. When the sequentially deposited layer is very thin (with a total thickness less than 2.5 nm in this study), a Ta_xMn_y single layer is formed by intermixing of the two layers, and then the Ta_xMn_y layer is oxidized by atmospheric exposure during the ex-situ DC sputtering of Cu layer. However, the 10-nm-thick Ta/Mn double layer remains deposited sequentially. As shown in the HR-TEM image of the inset in (b), the thin intermixed region is observed only at the interface between the Ta and Mn layers.
